# Sero-Prevalence and Risk Factors for Leptospirosis in Abattoir Workers in New Zealand

**DOI:** 10.3390/ijerph110201756

**Published:** 2014-02-05

**Authors:** Anou Dreyfus, Jackie Benschop, Julie Collins-Emerson, Peter Wilson, Michael G. Baker, Cord Heuer

**Affiliations:** 1 EpiCentre, Massey University, Private Bag 11 222, Palmerston North 4442, New Zealand; E-Mails: j.benschop@massey.ac.nz (J.B.); c.heuer@massey.ac.nz (C.H.); 2 Section of Epidemiology, Vetsuisse Faculty, University of Zurich, Zurich 8057, Switzerland; 3 Institute of Veterinary, Animal and Biomedical Sciences, Massey University, Private Bag 11 222, Palmerston North 4442, New Zealand; E-Mails: j.collins-emerson@massey.ac.nz (J.C.-E.); p.wilson@massey.ac.nz (P.W.); 4 Department of Public Health, University of Otago, P.O. Box 7343, Wellington 6242, New Zealand; E-Mail: michael.baker@otago.ac.nz

**Keywords:** abattoir, leptospirosis, *Leptospira borgpetersenii* sv. Hardjobovis, *Leptospira interrogans* sv. Pomona, microscopic agglutination test, sero-prevalence

## Abstract

Leptospirosis is an important occupational disease in New Zealand. The objectives of this study were to determine risk factors for sero-prevalence of leptospiral antibodies in abattoir workers. Sera were collected from 567 abattoir workers and tested by microscopic agglutination for *Leptospira interrogans* sv. Pomona and *Leptospira borgpetersenii* sv. Hardjobovis. Association between prevalence and risk factors were determined by species specific multivariable analysis. Eleven percent of workers had antibodies against Hardjobovis or/and Pomona. Workers from the four sheep abattoirs had an average sero-prevalence of 10%–31%, from the two deer abattoirs 17%–19% and the two beef abattoirs 5%. The strongest risk factor for sero-positivity in sheep and deer abattoirs was work position. In sheep abattoirs, prevalence was highest at stunning and hide removal, followed by removal of the bladder and kidneys. Wearing personal protective equipment such as gloves and facemasks did not appear to protect against infection. Home slaughtering, farming or hunting were not significantly associated with sero-prevalence. There is substantial risk of exposure to leptospires in sheep and deer abattoirs in New Zealand and a persisting, but lower risk, in beef abattoirs. Interventions, such as animal vaccination, appear necessary to control leptospirosis as an occupational disease in New Zealand.

## 1. Introduction

Leptospirosis is widespread in livestock in New Zealand (NZ). While in many, mainly subtropical countries, numerous animal hosts and *Leptospira* serovars survive in a complex ecological environment, the epidemiology of leptospirosis in NZ is based on just six endemic serovars. The two most frequent serovars in cattle, deer and sheep in NZ are *Leptospira borgpetersenii sv*. Hardjobovis (Hardjobovis) and *Leptospira interrogans sv.* Pomona (Pomona) [1,2]. Sixty percent of NZ deer herds, 92% of beef cattle herds, and 91% of sheep flocks had serological evidence of exposure to these serovars [3]. In NZ, livestock appear to be an important source of human leptospirosis, with farmers and meat workers being at a high risk [4]. Studies revealed that 62% of farmed deer [5] and 5.7% lambs sampled in abattoirs were sero-positive against Hardjobovis and/or Pomona [6]. Based on serology and culture, an abattoir worker was exposed to 5–9 deer or 5–26 lamb carcasses shedding *Leptospira* per day, hence presenting many opportunities for human infection [7].

NZ has a relatively high incidence of notified human cases among temperate developed countries [4] and a medium position for the Asia Pacific region [8]. Leptospirosis can result in severe human illness, but is rarely fatal in NZ. Notified human leptospirosis cases mainly represent severe clinical cases and milder forms remain under-reported [4]. The annual surveillance summary reports from 2006–2010 published by the Institute of Environmental Science and Research (ESR) [9] illustrate that cases were caused, in order of frequency, by serovars Hardjobovis, Ballum, and Pomona. From 2006 to 2010, 427 cases of leptospirosis were notified (86.4% laboratory confirmed by serology), giving an average annual rate of two cases per 100,000 population. 

The objectives of this study were to determine the prevalence of *Leptospira* in abattoir workers processing sheep, beef cattle or deer, to identify risk factors for sero-positivity related to occupational and non-occupational activities and to identify risk factors for probable leptospirosis and/or “flu-like-illness”.

## 2. Experimental Section

### 2.1. Study Design, Data Collection and Serological Testing

All procedures were approved by the Massey University Human Ethics Committee in 2009 [10]. Eight purposively selected abattoirs: four processing sheep, two beef and two deer, agreed to participate in a cross-sectional prevalence study on leptospirosis in meat workers. Two abattoirs were located in the South Island and six in the North Island of NZ Abattoir managers and supervisors, health and safety personnel, meat union representatives and workers were provided with information in meetings about the study objectives and procedures. Participation was voluntary and not based on random sampling. 

Between November 2009 and March 2010, blood was collected from participating meat workers by certified phlebotomists, and trained researchers conducted interviews. Information on work and non-work related risk factors including work positions for the last season, past work positions (for three former seasons), years worked in an abattoir, number of months working in the last and three previous slaughter seasons, personal protective equipment (e.g., safety glasses, gloves) worn in the current and previous work positions, lifestyle (hunting, farming, home slaughtering, outdoor activities in the last three years) and personal data such as age, gender, type of residence and ethnicity were recorded by questionnaire. Further, workers were asked whether they had been diagnosed with leptospirosis during their lifetime, whether they had had ‘flu-like’ symptoms over the past three years, how many days they were absent from work and whether they had received compensation. The questionnaire is in the supplementary materials. 

Ten mL of blood was collected with BD Vacutainer^®^ Plus tubes, stored between 4 °C and 10 °C in a mobile fridge, and couriered within 24 h in an icepack cooled Biocontainer^©^ to the Molecular Epidemiology and Public Health Laboratory (mEpilab) at Massey University in Palmerston North, NZ. After centrifugation of the clotted blood at 3,000 rpm for 6 min, the serum aliquots were transferred to duplicate cryovials and microtitre plates and stored at −80 °C. The microscopic agglutination test (MAT) was used to measure serum antibodies against Pomona and Hardjobovis at doubling dilutions from 1:24 to 1:1,536 as described previously [11]. The MAT was always performed by the same trained laboratory technician. 

### 2.2. Case Definitions

A *sero-positive case* was a participant with a titre of ≥1:48 against Pomona and/or Hardjobovis. 

A *probable leptospirosis case* had been diagnosed with leptospirosis of any serovar, by a health professional, at any point in time before the study period, on the basis of clinical symptoms with or without confirmation by laboratory test. 

A *case of* “*flu-like-illness*” was a worker who reported during the interview that an illness had occurred in the three years before the blood sample, based on the following symptoms: fever, headache, sweating, sore eyes, severe debility or sore muscles. Fever was not further defined, as participants were not able to remember the degree in Celsius. Workers were informed that the symptoms had to be severe enough that they felt like going home to rest.

### 2.3. Sample Size and Power Calculation

To detect a prevalence ratio of 2.5 with 80% power, a type I error of 5%, a prevalence of 10% in the exposed group, and an exposed to non-exposed ratio of 1/3, the required sample size was 280 study participants. To analyse the results for all abattoirs together, the sample size was doubled to take clustering within abattoir into account [12]. Hence a total required study size was 560 workers.

### 2.4. Data Analysis

Questionnaire information and serological test results were entered into an Access^©^ database and analyzed using Microsoft Excel^©^ and Stata 10 (StataCorp. 2007. *Stata Statistical Software: Release 10*. College Station, TX, USA) or SAS (SAS Institute Inc., Cary, NC, USA). Exploratory data analysis was conducted to validate the data and evaluate crude associations by using histograms, 2 × 2 tables and summary measures. 

The outcomes of interest were whether workers were sero-positive against Hardjobovis, Pomona or either serovar, and whether they had experienced “probable leptospirosis” or “flu-like-illness”. These outcome variables are shown as abattoir specific prevalence. The association between prevalence and risk factors was evaluated by chi-square analysis, separately for each slaughter species (sheep, cattle, and deer). The frequency, sero-status and time away from work of probable leptospirosis cases were described. Associations were analysed in two steps, firstly, by crude comparison of risk factors with outcomes, and secondly, by multivariate logistic regression. 

#### 2.4.1. Categories of Work Position and Personal Protective Equipment

Questions on work position were detailed, as this was likely the main potential risk factor for infection. Workers reported 153 work tasks, many of them being synonyms or overlapping between work positions. Depending on the abattoir and species slaughtered, some staff performed a wide range of activities in the abattoir, whereas others were occupied with a single task. In order to understand the risk of infection in different positions, we assigned work tasks to different work position categories with a similar exposure to urine or to organs of the urinary tract. With the aim to maximise statistical power, exposure groups with less than eight workers were merged with adjacent exposure groups. 

**Figure 1:**
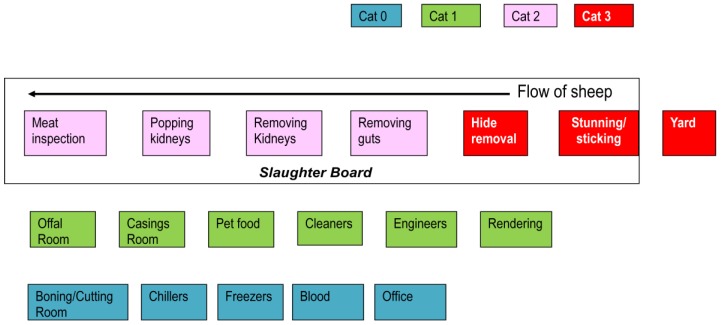
Schematic description of the various workplaces of workers in sheep abattoirs by category (colours) used in multivariable analysis.

Since the slaughter procedure is specific for each species, the work position categories were different for the three species. For sheep abattoirs, work positions were categorised into four, for deer into two and for beef cattle into four categories (Table 1). Figure 1 illustrates the work position categories for sheep: the reference (category 0) workers (blue) were from the “boning” room (where the carcass is cut into pieces), the “chillers”, “freezers”, “blood processing” or from the office; category 1 (green) workers were from the “offal”/“casing”/“pet food” rooms (where organs were handled) and hide processors, cleaners, renderers and engineers; category 2 (purple) included persons working in the middle and end of the slaughter board (where animals were opened, organs removed and carcasses were inspected); category 3 (red) were persons working in the yards (where animals were washed and waiting for slaughter) and at the beginning of the slaughter board (where animals were stunned, bled and hides were removed). The slaughter board contains workers from categories 2 and 3, but excludes workers from the “yards”. 

Work position categories in beef abattoirs also consisted of four categories, but differed from the composition of work tasks in sheep abattoirs. Category 3 included the yards, all workers on the slaughter board and meat inspectors, and category 2, workers in the “offal”/“casings”/“pet food” room. In category 1 were persons working in maintenance, cleaning or in the “plasma room” where blood is processed. Category 0 was the same as in sheep abattoirs. 

**Table 1:** Joint multivariable analysis of data from all plants: significant effects* on sero-prevalence of *Leptospira interrogans sv*. Pomona and/or *Leptospira borgpetersenii sv*. Hardjobovis in abattoir workers processing sheep (n = 325), deer (n = 56) and beef (n = 185) (November 2009–April 2010).

Species	Variable	Category	Adjusted OR	95% CI	*p*-value
Sheep	Work position	Boning, chillers, office	Ref.		
		Offal, pet food	6.5	1.4–29.8	0.017
		Gut removal, pulling kidneys	8.2	2.1–32.7	0.003
		Yards, stunning, pelting	10.4	2.8–38.8	<0.001
	Gender	Female	Ref.		
		Male	3.1	0.8–11.7	0.089*
	Years worked at meat plant	(Continuous)	1.1	1.0–1.1	0.011
	Meat plant	Sheep 1	Ref.		
		Sheep 2	4.5	1.2–16.3	0.022
		Sheep 3	6.3	1.8–22.4	0.004
		Sheep 4	2.1	0.7–6.3	0.201*
Deer	Work position	Boning, Chillers, Office	Ref.		
		Offal, pet food, gut removal, pulling kidneys, yards, stunning, pelting	12.7	1.3–120.6	0.027
	Wear facemask, or safety glasses	Never or sometimes	Ref.		
		Often or always	4.3	0.8–22.8	0.093*
Beef	Work position	Boning, chillers, office	Ref		
		Maintenance	2.0	0.3–23.4	0.59*
		Offal, pet food	3.1	0.5–20.6	0.25*
		Yards, stunning, pelting, gut, kidney removal & meat inspection	2.2	0.5–10.8	0.32*
	Age (years)	Continuous	1.1	1.0–1.2	0.02

Notes: The log likelihood values and p-values resulting from comparing the nested with the final model were for the sheep model −99.5; (*p* < 0.001), for the deer model −18.4 (*p* = 0.08) and beef model −34.3 (*p* = 0.006). The nested model included work position as single exposure variable and the Log likelihood of these nested models was −107.4 (sheep), −19.9 (deer) and −38.1 (beef); * Some effects were non-significant but have been included in this table based on their effect size and for comparison purposes.

Since most workers from deer abattoirs performed multiple tasks at the slaughter board, they could only be attributed to two work position categories: category 1 included workers at the slaughter board, in the yards and in the offal room and category 0 workers in boning or chilling rooms, and in the office, as in sheep and beef abattoirs. 

In the interview, study participants were asked about the type and frequency of Personal Protective Equipment (PPE) worn for every task at the meat plant. Type was defined as “facemasks” (mask with movable transparent protection shield covering the whole face), “safety (=goggles) or normal glasses”, “gloves on one or two hands” (made out of latex, or similar material or plastic) and “balaclava or beard mask”. Frequency categories were “always” (1), “often” (2), “sometimes” (3) or “never” (4). For increased power, exposure types were also analysed as two categories by merging categories 1 and 2, and 3 and 4.

#### 2.4.2. Multivariable Analysis

We developed models for each serovar separately, but since risk factors did not differ, we kept the model which combined the two serovars to increase the power. Due to differences in slaughter procedures and worker positions, separate multivariable logistic regression models were developed for each species. A forward selection method was chosen to evaluate exposure and confounding variables, starting with a null model with only an intercept included and then adding one variable at a time. A variable was allowed to enter if the Likelihood Ratio Test (LRT) was statistically significant at a *p*-value ≤0.20 and retained at *p* ≤ 0.05 or if their presence changed an exposure coefficient by more than 15% to account for bias. In addition, the following interaction terms were tested: “gender* wearing gloves”, “work position* gender”, “work position* wearing gloves”, “work position *wearing safety/normal glasses”, “wearing gloves* abattoir” and “wearing goggles or glasses* abattoir”. 

Continuous exposure variables were tested for linearity by plotting the Log odds of prevalence against quartiles of the variables “age”, “time worked at the current abattoir” and “time worked in the meat industry”. If the assumption was violated the quartiles were maintained. The Hosmer-Lemeshow statistic was used to test the distributional assumption and the Pseudo R-square to evaluate the overall model fit. Influential covariate patterns and leverage were examined using histograms of “Pearson Residuals”, “Hat matrix”, “Cook’s distance” and “DFBeta” [13].

## 3. Results

### 3.1. Participants, Slaughter Plants and Study Population

A total of 567 workers were interviewed and blood sampled. The participation proportions by plant ranged from 11%–61% (Table 2) and the number of participants by plant ranged from 21–112 (Table 3). The larger the workforce of an abattoir, the smaller was the participation rate. We estimated that about 30%–50% of the work force was present at the recruitment meetings. On average, participants worked 9.9 months in the slaughter season preceding the interview (*n* = 528), with 20 (3.8%) having worked 3 or fewer months. In 2006 there were 24,093 people employed in the meat industry (Meat Industry Association figures). The total work force at the eight study abattoirs (*n* = 2,661) represented approximately 11% of all workers in the meat industry in NZ assuming employment remained about constant.

**Table 2:** Number of workers, proportion recruited for the study, the species and total number processed and the regional origin of animals slaughtered in participating slaughter plants.

Abattoir	Total Number of Workers	Study Recruits (%)	Species Processed	Number of Animals Processed per Year	Regions of Animal Origin
Sheep 1	889	12	Lamb, mutton, bobby calves	1,797,809	Hawke’s Bay, Waikato, Wairarapa, Bay of Plenty, Northland
Sheep 2	378	26	Lamb, mutton, goats	600,469	Gisborne, Hawke’s Bay, Waikato, Bay of Plenty
Sheep 3	300	11	Lamb	780,000	Central Hawke’s Bay, East Coast, Wairarapa, Manawatu
Sheep 4	180	51	Lamb, mutton, bobby calves, goats	488,546	Wanganui, Manawatu, Taranaki, other
Deer 1	41	51	Venison	24,222	Canterbury
Deer 2	59	61	Farmed & feral ^a^ venison	41,055	South Waitaki River to Rakaia, North Canterbury
Beef 1	486	15	Beef cattle, dairy cows	93,837	East Coast, West Coast, Waikato, Bay of Plenty, Northland
Beef 2	328	34	Beef cattle	159,347	Taranaki, Waikato, Manawatu, Hawke’s Bay

Note: **^a^** Feral venison is integrated in the slaughter line after the stunning box. At this stage, the carcass has been opened and intestines and the urinary bladder have been removed, hence urine exposure is reduced.

### 3.2. Sero-prevalence and Antibody Titres

Sixty two study participants (10.9%, 95% CI 8.5%–13.9%) were sero-positive against either Hardjobovis or Pomona, of whom 10 (5.4%, 95% CI 2.7%–10.0%) were from beef (*n* = 185 workers), 10 (17.5%, 95% CI 9.1%–30.3%) from deer (*n* = 57 workers) and 42 (12.9%, 95% CI 9.6%–17.2%) from sheep abattoirs (*n* = 325 workers). The prevalence against Pomona and/or Hardjobovis in workers from the four sheep abattoirs ranged from 10%–31%, was 17 and 19% in the two deer abattoirs, and 5% in each of the two beef abattoirs. Twenty three sheep abattoir workers (7.1%) had antibodies against Pomona, 28 (8.6%) against Hardjobovis and 9 (2.8%) against both serovars. Three deer abattoir workers (5.3%) had antibodies against Pomona, eight (14%) against Hardjobovis and one (1.8%) against both serovars. Three (1.6%) beef abattoir workers had antibodies against Pomona, nine (4.9%) against Hardjobovis and two (1.1%) against both serovars (Table 3). 

**Table 3:** Sero-prevalence (%) and 95% confidence intervals (CI) of workers of eight abattoirs processing sheep, deer or beef with antibodies to *Leptospira interrogans sv*. Pomona (Pom), *Leptospira borgpetersenii sv.* Hardjobovis (Har) and to either serovar with a MAT titre cut-off of ≥1:48.

Abattoir	Participants	Prevalence (%)					
		Pom (%)	95% CI (%)	Har (%)	95% CI (%)	Either (%)	95% CI (%)
Sheep1	104	5	2–11	10	5–17	12	7–19
Sheep2	97	8	4–16	4	2–10	11	6–19
Sheep3	32	16	7–32	28	15–46	31	18–49
Sheep4	92	5	2–12	7	3–14	10	5–18
Deer1	21	5	1–27	19	7–41	19	7–41
Deer2	36	6	11–20	11	4–26	17	8–32
Beef1	73	3	1–10	4	1–12	5	2–14
Beef2	112	1	0–6	5	2–11	5	2–11
Total	567	5	3–7	8	6–10	11	8–14

The prevalence against Pomona and/or Hardjobovis in sheep plant workers working in the office, boning room or chillers was 2.2%, in the offal room was 11.4%, at the middle and end of the slaughter floor was 17.5%, and in those at the beginning of the slaughter floor was 27.9% (Table S1 in Supplementary Material). The prevalence against Pomona and/or Hardjobovis in deer plant workers working in the office, boning room or chillers was 2.9%, in the offal room, at the beginning, middle and end of the slaughter floor was 39.1% (Table S2 in Supplementary Material). The prevalence against Pomona and/or Hardjobovis in beef plant workers working in the office, boning room or chillers was 3.6%, in the offal room was 9.5%, in those at the beginning, at the middle and end of the slaughter floor was 5.6% (Table S3 in Supplementary Material). 

**Figure 2:**
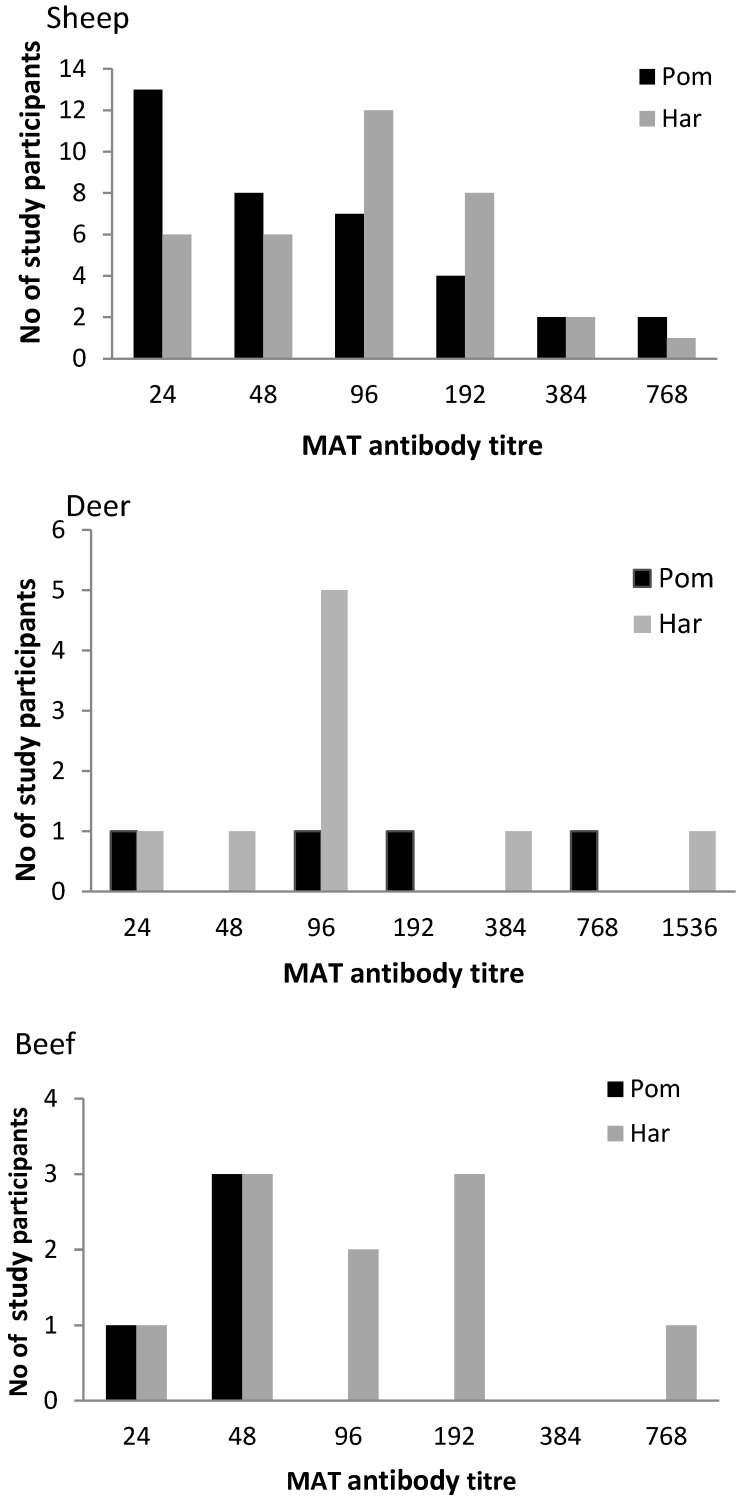
Frequency histogram showing the number of sero-positive study participants at each MAT titre to serovars *Leptospira interrogans sv*. Pomona (black) and *Leptospira borgpetersenii sv*. Hardjobovis (grey) in sheep (top), deer (middle) and beef abattoirs (bottom).

Reciprocal antibody titres against Pomona ranged from 0–768 in sheep and deer abattoir workers and from 24–48 in beef abattoir workers. Against Hardjobovis, titres ranged from 0–768 in sheep and beef abattoir workers and from 0–1,536 in deer abattoir workers (Figure 2). 

In three sheep, one deer and two beef abattoirs the prevalence of Hardjobovis titres in meat workers was higher than Pomona (*p* < 0.05), and in one deer and one sheep plant there was no statistically significant difference between Hardjobovis and Pomona prevalence (*p* > 0.05). 

### 3.3. Disease

Sixty workers had a history of probable leptospirosis while working in abattoirs between 1962 and 2010, of whom 27 were still sero-positive in this study. Forty probable leptospirosis cases were from sheep, five from deer and 15 from beef abattoirs. Twenty remembered the number of days being ill and away from work with the mean being 14.8 days (range 0–60 days). 

### 3.4. The Use of Personal Protective Equipment

The frequency of wearing PPE differed by species and abattoir. Whereas in sheep abattoirs 67% overall (range 42%–78% between abattoirs) of slaughter board, offal room and yard workers reported to (always or often) wear gloves on both hands, 17% did in deer (7%–33%) and 80% (77%–84%) in beef abattoirs. 

Seventy-one percent (range 11%–93% between abattoirs) of sheep, 43% (28%–67%) of deer and 38% (28%–51%) of beef slaughter board, offal room and yard workers reported to (always or often) wear safety/normal glasses and 12%, 9% and 4%, respectively, to wear facemasks. On the slaughter board itself the requirement for wearing gloves and glasses varied between abattoirs. While 95% of workers in the stunning area of one sheep plant (no. four) reported wearing glasses or facemasks, 7% did in another sheep plant (no. three). There was, however, no significant difference in wearing glasses or facemasks in areas where kidneys were removed and offal was handled. 

### 3.5. Risk Factors for Sero-prevalence in Sheep Plants

The crude associations between exposure variables and sero-positivity against Pomona and/or Hardjobovis for sheep abattoir workers are shown in the Supplementary Material (Tables S1 and S4). Compared with the workers working in the office, boning room or chillers (reference group), meat workers working in the offal room (OR = 5.8), removing kidneys (OR = 9.6) or stunning/pelting (OR = 17.4) had a higher odds of positivity. Being male (OR = 6.4), working in sheep plant 3 (OR = 3.5), having had probable leptospirosis (OR = 10.3), always or often wearing a facemask (OR = 2.8), always wearing safety glasses (OR = 2.5) increased the odds of positivity in meat workers. Moreover, the longer they worked at the current plant, the more likely they were positive against *Leptospira*. 

The variables remaining in the final logistic regression model were “work position”, “gender”, “years worked in the meat plant” and “meat plant” (Table 1). Compared with the workers working in the office, boning room or chillers (reference group), meat workers working in the offal room had 6.5 (95% CI 1.4–29.8, *p* = 0.017) times the odds of being sero-positive against Pomona and/or Hardjobovis, and workers at the middle and end of the slaughter floor had 8.2 times the odds (95% CI 2.1–32.7, *p* = 0.003) and those at the beginning of the slaughter floor 10.4 times the odds (95% CI 2.8–38.8, *p* < 0.001). Men were 3.1 (95% CI 0.8–11.7, *p* = 0.09) times as likely to be sero-positive as women once controlled for the effect of work position. For every year working in the meat plant the odds of being sero-positive increased 1.08-fold, suggesting an eight percent increase in risk for each year of working in the plant (*p* = 0.01). Compared to working in sheep plant 1, working in sheep plants 2, 3 and 4 increased the odds of sero-positivity by 4.5 (*p* = 0.02), 6.3 (*p* = 0.004) and 2.1 (*p* = 0.2) respectively. The variables “gender” and “wearing safety/normal glasses” were confounding variables as they reduced the adjusted OR for sero-positivity of work position category 3 (stunning/pelting) by more than 15%. However, “gender” was marginally significantly associated with sero-status (*p* = 0.09) whereas “wearing safety/normal glasses” was not (*p* = 0.7). None of the tested interactions were significant. Maybe females were more careful not to get splashed with urine when working in the stunning/pelting area. 

### 3.6. Risk Factors for Sero-prevalence in Deer Abattoirs

Crude associations between exposure variables and sero-positivity against Pomona and/or Hardjobovis for deer abattoir workers are shown in the Supplementary Material (Tables S2 and S5). Work position, the use of PPE and having had probable leptospirosis were risk factors for being sero-positive (OR > 1; *p* ≤ 0.05) in simple, bivariable analyses.

After adjusting for PPE (facemask or safety/normal glasses) in the multivariable logistic regression analysis, study participants working at the slaughter board or offal room were 12.7 (95% CI 1.33–120.6, *p* = 0.027) times as likely to be sero-positive against Pomona and/or Hardjobovis as were participants working in the office, boning room or chillers. Sero-prevalence for workers wearing PPE was 4.24 (95% CI 0.79–22.82, *p* = 0.09) as high as those who did not wear PPE, a marginally significant finding (Table 1). However, the low sample size of 57 deer abattoir workers provided limited statistical power for the risk factor analysis. 

The inclusion of PPE (“wearing a facemask or safety/normal glasses”) in the multivariable model reduced the crude OR of the high-risk work position from 21.1 to an adjusted OR of 12.7, presumably due to a confounding effect of workers in high-risk work positions wearing facemasks or safety glasses more often than workers in the boning room or office (43.5% *vs*. 5.9%, *p* = 0.001).

Since no female participant was sero-positive, a model including gender and work position failed to converge due to only 9 female workers in the two participating deer plants. None of the tested interactions were significant.

### 3.7. Risk Factors for Sero-prevalence in Beef Abattoirs

Crude associations between exposure variables and sero-positivity against Pomona and/or Hardjobovis for beef abattoir workers are shown in the Supplementary Material (Tables S3 and S6). Crude odds of being sero-positive increased linearly with age (*p* < 0.05) and having had probable leptospirosis was positively associated with being sero-positive (<0.001). However, none of the work positions was associated with an increased or decreased risk of being sero-positive. 

In the multivariable model, aging by one year increased the odds of being sero-positive 1.09-fold, suggesting an increase by 9% of the baseline prevalence for each year of age (*p* = 0.02). As in the deer model, none of the tested female workers in the beef abattoirs were sero-positive despite 45 female participants, thus a model with gender failed to converge. Work position and abattoir were not significantly associated with sero-positivity (Table 1).

Diagnostics of the final multivariable models indicated a good fit of the data for all three species. Even though outliers were identified, they did not impact on any of the inferences.

## 4. Discussion and Conclusions

### 4.1. Sero-prevalence

The serological prevalence to serovars Hardjobovis or Pomona was highest among workers from the four sheep abattoirs (10%–31%), followed by two deer abattoirs (17%, 19%) and two beef abattoirs (5%, 5%). The finding of a high prevalence in sheep plant workers contradicts an established view that sheep were not an exposure source for people [14]. Therefore, the public health importance of sheep may have been underestimated in the past. 

A possible reason for the difference between sheep and beef abattoir worker prevalences is in variable slaughter procedures and species peculiarities. During interviews, participants reported that sheep often urinate spontaneously when stunned, whereas this was rarely observed in beef cattle. Exposure is likely to increase due to stunned sheep touching down on the platform where urine of sheep accumulates. In addition, sheep and deer plants process more animals per day than beef plants. This and the fact that carcass prevalence is expected to be lower in dairy cattle due to vaccination, sheep plant workers are likely to be more exposed to *Leptospira* than beef plant workers [15,16]. The perception that sheep are not an important source of leptospirosis may also have contributed to less rigorous application of safety precautions in this environment.

While there was a negative correlation between compliance in wearing gloves and glasses or facemasks and prevalence in slaughter floor workers of sheep plants, in deer and beef plant workers there was no such correlation (hence plants with a higher prevalence did not have a lower compliance in wearing PPE). That some plants achieved better compliance with PPE policy than others may therefore not have accounted for prevalence differences across plants.

The prevalence estimates were not entirely representative for the total occupational workforce as enrolment in the study was voluntary. The multivariable analysis of the data revealed, that work position had an effect on participation: workers from more exposed work positions were more likely to participate. Other confounders did not seem to be affected by the biased sampling fraction. The sampling bias was addressed in a parallel analysis, in which the distribution of workers in different work positions in the sample was weighted by the distribution in the entire workforce by direct standardisation [17]. Crude and bias-adjusted prevalence of workers for the three slaughter species was: 16% *vs.* 11% for sheep, 18% *vs.* 11% for deer and 5% *vs.* 5% for beef. Thus apart of a considerable over estimate for workers of deer plants, the overall bias was in reasonable range. Supplementary Material (Tables S1, S2 and S3) provides prevalence estimates for each work position for each slaughter species ranging from 2% to 39%.

Conclusions on risk factors (ORs) from the multivariable logistic regression analysis, where working area was included as a covariate, were not affected by the sampling bias.

Even though the ability of the MAT to distinguish between serovars has been questioned [18,19], it is unlikely that this was the case for Hardjobovis and Pomona in this research, as the prevalent serovars in NZ belong to different serogroups apart from Hardjobovis and *Leptospira borgpetersenii sv.* Balcanica (Balcanica) [14]. Further, several studies have been conducted in NZ in recent years, where serovars determined by serology had been also confirmed by direct methods. For example, MAT serology and serovar isolates had good kappa agreement by DNA sequencing results [20,21]. 

In this study we measured prevalence of exposure, not clinical disease and therefore chose a lower MAT titre cut-off (1:48), than studies intending to detect clinical leptospirosis (1:≥100). Even though Faine *et al.* [11] and Shivakumar *et al.* [22] suggested a titre cut-off of 1:50 to test exposure to *Leptospira* spp., they did not specify the sensitivity and specificity of the MAT for the given cut-off. In the literature search, we could not find any specification of sensitivity and specificity of the MAT to estimate prevalence of exposure for a given cut-off. In a study evaluating the MAT sensitivity and specificity of acute (MAT cut-off 1:100) and convalescent (MAT cut-off not mentioned) sera in an urban setting in Brazil [23], the MAT testing of convalescent sera had a sensitivity of 91%–100% and specificity of 94%–100%. If we assumed that the MAT in our study had a 91% sensitivity and 94% specificity, the tested prevalence in meat plants was likely under-estimated. However, since we used a MAT titre cut-off of 1:48 and tested for the serovars Hardjobovis and Pomona, which are less likely to be encountered in an urban setting, where Copenhageni is predominant [23], it is possible that the sensitivity and specificity of the MAT in NZ are not the same as in Brazil. 

NZ studies on leptospirosis prevalence in meat workers were conducted in multispecies abattoirs slaughtering sheep, beef and sometimes pigs in the 1980s, and revealing a prevalence between 0%–2.7% against Hardjobovis and 0.8%–8.9% against Pomona (MAT titre cut-off 1:24) [24,25]. A recently conducted study in a sheep abattoir revealed in 242 meat workers a prevalence of 9.5% against Hardjobovis or Pomona (MAT titre cut-off 1:24) [26]. In our study, workers from the four sheep abattoirs had an average prevalence of leptospiral titres (Hardjobovis or Pomona) of 10%, 11%, 12% and 31%, from the two deer abattoirs 17% and 19% and the two beef abattoirs 5% and 5%. A precise comparison between the older Blackmore *et al.* studies having tested workers from multispecies plants and this abattoir study is difficult because we tested workers from single species plants, where *Leptospira* prevalence can be associated with exposure to one species. Nevertheless, the prevalence in sheep and cattle meat workers seems to have increased, especially since we used a higher MAT cut-off of 1:48 to define a sero-positive test result. 

In six of eight meat plants serovar Hardjobovis was more prevalent than Pomona in meat workers (*p* < 0.05). This serovar distribution of Hardjobovis and Pomona is as well described in slaughter lambs and deer [5,27,28]. However, the higher prevalence of Hardjobovis in meat workers may be due to longer antibody titre duration of Hardjobovis and not because of a higher infection risk [29].

### 4.2. Probable Leptospirosis

A previous experience of probable leptospirosis was strongly predictive for the presence of antibody. It is a common finding that antibodies from clinical leptospirosis persist for many years [24,30]. However, that these workers were serologically positive up to 20–30 years after the clinical episode may be attributable to continued high exposure and multiple re-infection rather than antibody persistence *per se*. 

Despite 43.6% (247/567) workers reporting “flu-like illness” during the past 36 months, there was no statistically significant association between “flu-like illness” and leptospirosis sero-positivity. This would suggest that almost all infections with *Leptospira* were asymptomatic. However, the time period of 36 months in which “flu-like illness” occurred was most likely too long to measure an association between “flu-like illness” and positivity. 

### 4.3. Risk Factors for Sero-positivity

This study demonstrated that work position was the strongest risk factor for sero-positivity with Pomona and/or Hardjobovis in sheep and deer abattoir workers. The higher prevalence in workers at the beginning of the slaughter board and the gradual reduction along the slaughter line in sheep plants is consistent with a study conducted two years earlier in one of the sheep plants of this study [31]. Urine splashing due to stunning and subsequent contamination of pelts and carcasses are thought to be causes for infection, which may be difficult to control while handling carcasses. The prevalence of workers half way down the slaughter board may be attributable to exposure to *Leptospira* from organs of the genital-urinary tract. Evisceration may therefore pose another risk of infection, either when organs are removed from the carcass, processed or inspected. Even though exposure to shedding may even be higher at evisceration and offal handling than at the beginning of the slaughter line [7], the time and place of exposure is more predictable in these positions and workers can clean hands more frequently than at the physically challenging and injury prone positions at the head of the slaughter board. Persons in the other processing areas (boning room, chillers) or in the office had little or no exposure to urine and were therefore less likely to get infected. Controlling leptospirosis in livestock aside, control measures targeting the most high risk components of the abattoir process would be likely to have the greatest protective impact. Hence, measures to contain urine during stunning and urinary tract tissues should be investigated. The removal of the platform sheep land on after stunning, may be a useful control measure, as each sheep landing on the platform might get contaminated with urine from former sheep, increasing the risk of spreading contaminated urine further down the line. 

The use of PPE appeared to increase rather than reduce the risk of sero-positivity. In the multivariable model PPE had a marginally significant positive OR (*p* = 0.08) in deer workers. This may have biological plausible reasons. For example, workers may wipe their eyes to remove the sweat more often with their contaminated hands when wearing glasses or facemasks. Meat workers stated during interviews that they often had to lift up their safety glasses or masks to remove sweat and fog, and restore visibility. Moreover, water accumulating in latex gloves moistens the skin with a possible consequence of reducing the natural outer defence layer of the skin.

The finding that the PPE may not be protective warrants further investigation. Using PPE for most tasks is not comfortable, and if workers are mandated to wear protective gear, it seems reasonable to expect a benefit from doing so. This finding supports use of other means of protection, notably vaccination of farmed livestock. Since vaccination of dairy cows commenced in the 1980s, the incidence of notified human leptospirosis cases in the farming industry decreased from 234 annual cases per 100,000 to 90 per 100,000 [2,4,32]. Whereas a large proportion of dairy farmers are known to vaccinate their stock against leptospirosis and the NZ pig industry introduced compulsory vaccination of pig herds [33] less than 10% of deer, sheep or beef farmers are currently using vaccination [34,35]. Vaccination also has the potential to protect farmers and farm workers, veterinarians and veterinarian technicians, shearers, truck drivers, artificial insemination technicians and home butchers who are also at risk of infection [15,21,36,37,38]. No registered vaccine is currently available for humans in NZ.

In our analysis, male workers of sheep plants were more likely to be sero-positive than females. This association was not confounded by age, hunting, home slaughter or work position and there was no difference in the frequency of wearing PPE in exposed work position categories between men and women. Females were as likely to get exposed to urine as men within the work position categories with high urine exposure. Moreover, there was no interaction between gender and work position. Therefore, the evidence suggested that the prevalence difference between males and females was a real gender effect. This difference could be caused by behavioural factors, such as subtle variations in use of PPE and handwashing, though more research would be needed to investigate such hypotheses. 

The identification of worker age as a risk factor in beef plants could not be explained with increased exposure over time as the variable “time worked in the industry” was not statistically significant in the model. A possible reason could be changes in the immune system with age. Categories of age were not associated with work position categories. Therefore, the effect of age on prevalence was not confounded by work position. 

Other exposure factors, such as home slaughtering, farming, hunting or smoking were all unrelated to prevalence in the multivariable analysis, regardless of species processed. This partially contrasts the findings of a previous study in one sheep plant, where home slaughtering was found to be a risk factor [31]. Our study had sufficient statistical power to identify strong risk factors (OR > 2.5) and we studied four instead of one sheep plant and therefore believe that the results of our study had more power and was more representative. 

A sero-positive case was a study participant with a titre of ≥1:48 against Pomona and/or Hardjobovis. The reason for not distinguishing between serovars was that, in NZ, beef, sheep and deer were known to be most frequently infected with either or both serovars, and there is no evidence that human exposure factors would be different for the two serovars [14,28,39]. Further, compiling both serovars together in one outcome increased the power of the study. We ran the multivariable analysis for Pomona and Hardjobovis prevalence separately and were assured that risk factors for the two serovars did not differ. The titre cut-off of 1:48 was recommended to determine exposure to leptospires, but not for clinical disease [11,22]. 

The conclusions are as following. This study demonstrated that workers from the four sheep abattoirs had an average prevalence of leptospiral titres (Hardjobovis or Pomona) of 10-31%, and from two deer abattoirs of 17% and 19%. In contrast, prevalence was lower in workers processing cattle (5%). Antibodies were more frequently found against serovar Hardjobovis (61%) than Pomona (39%), and this was similar to the serovar difference reported from livestock. The strongest risk factor for sero-prevalence of workers in sheep and deer abattoirs was work position. For participants from sheep plants, prevalence was highest at the beginning of the slaughter board, lower in those working where activities involved the removal of high risk material (guts, bladder, and kidneys), even lower in those in the offal/pet food area, and lowest in those in the boning room or office. This finding raises the hypothesis that stunning and pelting constitutes a higher exposure risk than direct contact with internal viscera like kidneys, the prime tissue reservoir for *Leptospira*. 

The data suggested that wearing personal protective equipment such as gloves, facemasks, safety/normal glasses or a balaclava did not protect against infection. Hence this study raises questions about best practice use of PPE. Vaccination of deer, sheep and beef herds needs to be considered if occupational transmission of leptospirosis is to be controlled. Non work-related risk factors, such as home slaughtering, farming or hunting were not significantly associated with prevalence in this study. About 11% of workers reported to have experienced probable leptospirosis during a median period of five years prior to the study, confirming the occupational health significance of this disease. The incidence of mild or severe clinical leptospirosis in abattoir workers and the economic impact remains unknown and warrants clarification by further research. 

## Supplementary Files

Supplementary File 1 Supplementary Information (PDF, 567 KB)
