# Supplementary material for: Sero-Prevalence and Risk Factors for Leptospirosis in Abattoir Workers in New Zealand
Source: Int J Environ Res Public Health. 2014 Feb 5;11(2):1756–75. doi: 10.3390/ijerph110201756 (PMC3945566; doi:10.3390/ijerph110201756)
Supplement: Supplementary File 1 — Supplementary Information (PDF, 567 KB) [file ijerph-11-01756-s001.pdf]

Sero-Prevalence and Risk Factors for Leptospirosis in Abattoir Workers in New Zealand

Table S1. Frequencies of work-related exposure variables and their unconditional association with sero-prevalence of *Leptospira interrogans sv*. Pomona and/or *Leptospira borgpetersenii sv*. Hardjobovis in sheep plant workers (n = 325) blood sampled and interviewed from January–April 2010.

Variable	Category	% Workers (n)	Sero-prevalence %	Crude OR	95% CI	*p*-value
Work position	Boning, chillers, office	42.5 (138)	2.2	Ref.		<0.001 [2]
	Offal, pet food	13.5 (44)	11.4	5.8	1.3–25.2	0.02
	Gut & kidney removal, meat inspection	17.5 (57)	17.5	9.6	2.5–36.3	0.001
	Yards, stunning, pelting	26.5 (86)	27.9	17.4	5.0–60.0	<0.001
Wear gloves on both hands	never	29.2 (95)	13.7	Ref.		0.99 [2]
	sometimes	12 (39)	12.8	0.9	0.3–2.8	0.894
	often	2.8 (9)	11.1	0.8	0.1–6.8	0.829
	always	56 (182)	12.6	0.9	0.4–1.9	0.806
Wear safety glasses	never	37.9 (123)	7.3	Ref.		0.11 [2]
	sometimes	12.9 (42)	16.7	2.5	0.9–7.3	0.085
	often	6.2 (20)	15.0	2.2	0.5–9.1	0.261
	always	43.1 (140)	16.4	2.5	1.1–5.6	0.028
Wear a facemask	never or sometimes	93.2 (303)	11.9	Ref.		
	often or always	6.8 (22)	27.3	2.8	1.0–7.6	0.04
Wear a balaclava	never or sometimes	69.5 (226)	11.1	Ref.		
	often or always	30.5 (99)	17.2	1.7	0.9–3.2	0.1
Months worked at current plant	≤60	34.2 (111)	6.3	Ref.		0.04[2]
	>60 - 108	20.9 (68)	13.2	2.3	0.8–6.4	0.122
	> 108-192	20.9 (68)	16.2	2.9	1.0–7.8	0.039
	>192	24 (78)	19.2	3.5	1.4–9.1	0.009
Months worked in meat industry [1]	≤60	28.3 (73)	6.8	Ref.		0.25[2]
	>60 - 123	22.9 (59)	16.9	2.8	0.9–8.6	0.078
	> 123-288	27.5 (71)	15.5	2.5	0.8–7.6	0.108
	>288	21.3 (55)	14.5	2.3	0.7–7.5	0.162
Smoking at work	No	71.7 (231)	13.9	Ref.		
	Yes	28.3 (91)	11.0	0.8	0.4–1.6	0.493
Abattoir	Sheep plant 1	32 (104)	11.5	Ref.		0.04 [2]
	Sheep plant 2	29.9 (97)	11.3	1.0	0.4–2.3	0.965
	Sheep plant 3	9.9 (32)	31.3	3.5	1.3–9.1	0.011
	Sheep plant 4	28.3 (92)	9.8	0.8	0.3–2.1	0.692

Notes: [1] n = 258; [2] *p*-value of the LRT for all exposure categories combined.

Table S2. Frequencies of work related exposure variables and their unconditional association with sero-prevalence of *Leptospira interrogans sv*. Pomona (Pom) and/or *Leptospira borgpetersenii sv*. Hardjobovis (Har) in deer plant workers (n = 57) blood sampled and interviewed in November 2009.

**Variable**	**Category**	**% Workers (n)**	**Sero-Prevalence %**	**Crude OR**	**95% CI**	***p*-value**
Work position	Boning, chillers, office	59.7 (34)	2.9	Ref.		
	Offal, pet food, gut & kidney removal, yards, stunning, pelting	40.4 (23)	39.1	21.2	2.4–183.7	0.006
Wear gloves on both hands	never	73.7 (42)	21.9			0.65 [2]
	sometimes	5.3 (3)	10.0	0.4	0.0–3.7	0.416
	often		0.0			
	always	21.1 (12)	15.4	0.6	0.1–3.6	0.624
Wear safety glasses	never	73.7 (42)	7.1			0.004 [2]
	sometimes	5.3 (3)	33.3	6.5	0.4–94.1	0.17
	often		0.0			
	always	21.1 (12)	50.0	13.0	2.5–66.4	0.002
Wear a facemask	never or sometimes	96.5 (55)	16.4	Ref.		
	often or always	3.5 (2)	50.0	5.1	0.3–89.5	0.264
Wear a balaclava	never or sometimes	91.2 (52)	17.3	Ref.		
	often or always	8.8 (5)	20.0	1.2 [3]	0.1–12.0	0.88
Months worked at current plant	≤60 months	50 (13)	15.4			
	>60–108 months	15.4 (4)	0.0			
	>108–192 months	34.6 (9)	33.3			
	>192 months		0.0			
Months worked in meat industry [1]	≤60 months	21.1 (12)	0.0	[3]		
	>60–123 months	26.3 (15)	33.3			
	>123–288 months	24.6 (14)	14.3			
	>288 months	28.1 (16)	18.8			
Smoking at work	No	58.9 (33)	18.2	Ref.		
	Yes	41.1 (23)	17.4	0.9	0.2–3.8	0.939
Abattoir	Deer plant1	36.8 (21)	19.0	Ref.		0.9 [2]
	Deer plant2	63.2 (36)	16.7	0.8	0.2–3.4	0.82

Notes: [1] n = 258; [2] *p*-value of the LRT for all exposure categories combined; [3] model did not run, due to data sparsity issues.

Table S3. Frequencies of work related exposure variables and their unconditional association with sero-prevalence of *Leptospira interrogans sv*. Pomona (Pom) and/or *Leptospira borgpetersenii sv*. Hardjobovis (Har) in beef plant workers (n = 185) blood sampled and interviewed from January–April 2010.

**Variable**	**Category**	**% Workers (n)**	**Sero-Prevalence %**	**Crude OR**	**95% CI**	***p*-value**
Work position	Boning, chillers, office	44.9 (83)	3.6	Ref.		0.77 [3]
	Maintenance	5.4 (10)	10	3.0	0.3–31.6	0.368
	Offal, pet food	11.4 (21)	9.5	2.8	0.4–18.0	0.276
	Yards, stunning, pelting, gut & kidney removal, meat inspection	38.4 (71)	5.6	1.6	0.3–7.4	0.552
Wear gloves on both hands [1]	never	20.1 (37)	2.7	Ref.		0.25 [3]
	sometimes	13 (24)	0.0	[4]		
	often	2.7 (5)	0.0	[4]		
	always	64.1 (118)	7.6	2.8	0.4–24.3	0.309
Wear safety glasses	never	66 (122)	5.7	Ref.		0.47 [3]
	sometimes	4.3 (8)	0.0	[4]		
	often	2.7 (5)	20.0	4.1	0.4–41.8	0.233
	always	27 (50)	4.0	0.7	0.1–3.4	0.644
Wear a facemask	never or sometimes	97.8 (181)	5.0	Ref.		
	often or always	2.2 (4)	25.0	6.4	0.6–67.5	0.124
Wear a balaclava [1]	never or sometimes	92.4 (171)	5.8	[4]		
	often or always	7.6 (14)	0.0			
Months worked at current plant	<61 months	27 (50)	2.0	Ref.		0.12 [3]
	61–108 months	24.3 (45)	0.0	[4]		
	109–192 months	21.6 (40)	12.5	7.0		0.082
	>192 months	27 (50)	8.0	4.3	0.5–39.5	0.202
Months worked in meat industry [2]	<61 months	22.7 (42)	2.4	Ref.		0.19 [3]
	61–123 months	26.5 (49)	0.0	[4]		
	124–288 months	22.2 (41)	12.2	5.7	0.6–51.0	0.12
	>288 months	28.7 (53)	7.5	3.3	0.4–31.1	0.288
Smoking [2]	No	73.4 (127)	7.1	Ref.		
	Yes	26.6 (46)	2.2	0.3	0.0–2.4	0.248
Abattoir	Beef plant1	39.5 (73)	5.5	Ref.		
	Beef plant2	60.5 (112)	5.4	1.0	0.3–3.6	0.971

Notes: [1] n = 184; [2] n = 173; [3] *p*-value of the LRT for all exposure categories combined; [4] model did not converge, category dropped, due to sparse data.

Table S4. Frequencies of clinical, demographic and non-work related exposure variables and their unconditional association with sero-prevalence of *Leptospira interrogans sv*. Pomona and/or *Leptospira borgpetersenii sv*. Hardjobovis in sheep plant workers (n = 325) blood sampled and interviewed from January–April 2010.

**Variable**	**Category**	**% Workers (n)**	**Sero-Prevalence %**	**Crude OR**	**95% CI**	***p*-value**
Probable	No	87.7 (285)	8.1	Ref.		
Leptospirosis [1]	Yes	12.3 (40)	47.5	10.3	4.8–21.9	<0.001
Had	No	63.4 (206)	13.1	Ref.		
flu-like-illness	Yes	36.6 (119)	12.6	0.9	0.5–1.9	0.88
Gender	Female	29.5 (96)	3.1	Ref.		
	Male	70.5 (229)	17.0	6.4	1.9–21.1	0.003
Age	≤ 37	25.5 (83)	14.5	Ref.		0.5 [5]
	>37, ≤48	28 (91)	8.8	0.6	0.2–1.5	0.246
	>48, ≤55	22.2 (72)	12.5	0.8	0.3–2.1	0.723
	>55	24.3 (79)	16.5	1.2	0.5–2.7	0.725
Hunting any	No	83.1 (270)	13.3	Ref.		
Species [2]	Yes	16.9 (55)	10.9	0.8	0.3–2.0	0.626
Hunting pigs,	No	85.9 (279)	13.3	Ref.		
deer or feral goats	Yes	14.2 (46)	10.9	0.8	0.3–2.1	0.655
Farming [3]	No	84.6 (275)	13.8	Ref.		
	Yes	15.4 (50)	8.0	0.5	0.2–1.6	0.26
Ethnicity	NZ European	33.9 (110)	8.2	Ref.		0.19 [5]
	NZ Maori	57.5 (187)	15.5	2.0	0.9–4.5	0.072
	Other	8.6 (28)	14.3	1.9	0.5–6.6	0.33
Slaughtering at	No	69.5 (226)	10.6	Ref.		
home [4]	Yes	30.5 (99)	18.2	1.9	1.0–3.6	0.06

Notes: [1] was not included in the multivariable model, as it was an intermediate variable between exposure and antibody level; [2] pigs, deer, goats, birds, rodents, possums; [3] pigs, goats, sheep, beef cattle, alpaca or deer; [4] sheep, goats, pigs, beef or deer; [5] *p*-value of the LRT for all exposure categories combined.

Table S5. Frequencies of clinical, demographic and non-work related exposure variables and their unconditional association with sero-prevalence of *Leptospira interrogans sv.* Pomona (Pom) and/or *Leptospira borgpetersenii sv*. Hardjobovis (Har) in deer plant workers (n = 57) blood sampled and interviewed in November 2009.

**Variable**	**Category**	**% Workers (n)**	**Sero-Prevalence %**	**Crude OR**	**95% CI**	***p*-value**
Probable leptospirosis [1]	No	91.2 (52)	11.5	Ref.		
	Yes	8.8 (5)	80	30.7	2.9–321.8	0.004
Had flu-like-illness	No	38.6 (22)	22.7	Ref.		
	Yes	61.4 (35)	14.3	0.7	0.1–2.2	0.42
Gender	Female	15.8 (9)	0.0	Ref.		
	Male	84.2 (48)	20.8	[6]		
Age	<32	26.3 (15)	20.0	Ref.		0.93 [5]
	32–42	24.6 (14)	14.3	0.7	0.1–4.7	0.685
	43–47	24.6 (14)	21.4	1.1	0.2–6.6	0.924
	>47	24.6 (14)	14.3	0.7	0.9–4.7	0.685
Hunting any species [2]	No	63.2 (36)	13.9	Ref.		
	Yes	36.8 (21)	23.8	1.9	0.5–7.7	0.347
Hunting pigs, deer or feral goats	No	70.2 (40)	12.5	Ref.		
	Yes	29.8 (17)	29.4	2.9	0.7–11.8	0.135
Farming [3]	No	80.7 (46)	17.4	Ref.		
	Yes	19.3 (11)	18.2	1.1	0.2–5.8	0.951
Ethnicity	NZ European	87.7 (50)	18.0	Ref.		
	NZ Maori	7 (4)	25.0	0.8 [7]	0.1–7.1	0.809
	Other	5.3 (3)	0.0			
Slaughtering at home [4]	No	64.9 (37)	18.9	Ref.		
	Yes	35.1 (20)	15.0	0.8	0.2–3.3	0.711

Notes: [1] Not included in the multivariable model, as it was an intermediate variable between exposure and antibody level, hence not a confounder; [2] pigs, deer, goats, birds, rodents, possums; [3] pigs, goats, sheep, beef cattle, alpaca or deer; [4] sheep, goats, pigs, beef & or deer; [5] *p*-value of the LRT for all exposure categories combined; [6] The category group "female" had no *Leptospira* sero-positive observations; [7] ethnicity: category 2 and 3 were collapsed into category 2.

Table S6. Frequencies of clinical, demographic and non-work related exposure variables and their unconditional association with sero-prevalence of *Leptospira interrogans sv.* Pomona and/or *Leptospira borgpetersenii sv*. Hardjobovis in beef plant workers (n = 185) blood sampled and interviewed from January–April 2010.

**Variable**	**Category**	**% Workers (n)**	**Sero-Prevalence %**	**Crude OR**	**95% CI**	***p*-value**
Probable leptospirosis [1]	No	91.9 (170)	1.8	Ref.		
	Yes	8.1 (15)	46.7	48.7	10.6–224.3	<0.001
Had flu-like-illness	No	49.7 (92)	7.6	Ref.		
	Yes	50.3 (93)	3.2	0.4	0.1–1.6	0.2
Gender	Female	24.3 (45)	0.0	[7, 8]		0.06 [6]
	Male	75.7 (140)	7.1			
Age	≤34	25.4 (47)	0.0	[7,8]		0.1 [6]
	>34, ≤48	26.5 (49)	4.1			
	>48, ≤56	24.3 (45)	6.7			
	>56	23.8 (44)	11.4			
Hunting all species [2]	No	86 (159)	5.0	Ref.		
	Yes	14.1 (26)	7.7	1.6	0.3–7.9	0.581
Hunting pigs, deer or feral goats	No	88.1 (163)	5.5	Ref.		
	Yes	11.9 (22)	4.5	0.8	0.1–6.8	0.85
Farming [3]	No	84.3 (156)	5.8	Ref.		
	Yes	15.7 (29)	3.4	0.6	0.1–4.8	0.616
Ethnicity	NZ European	41.1 (76)	5.3	Ref.		0.07 [6]
	NZ Maori	44.9 (83)	2.4	0.4	0.1–2.5	0.357
	Other	14.1 (26)	15.4	3.3	0.8–14.2	0.113
Slaughtering at home [4]	No	72.4 (134)	6.0	Ref.		
	Yes	27.6 (51)	3.9	0.6	0.1–3.1	0.585

Notes: [1] was not included in the multivariable model, as it was an intermediate variable between exposure and antibody level; [2] pigs, deer, goats, birds, rodents, possums; [3] pigs, goats, sheep, beef cattle, alpaca & or deer; [4] sheep, goats, pigs, beef & or deer, [5] is an intermediate variable; [6] *p*-value of the LRT for all exposure categories combined; [7] the category groups "female" and "age group 1" had no *Leptospira* sero-positive observations; [8] model did not run, due to data sparsity issues.

Study on Leptospirosis among Abattoir Workers

Participant Questionnaire

The research team appreciates your involvement in this study of leptospirosis and is committed to privacy of all personal information.

The information from this questionnaire will help us to assess the risk of contracting leptospirosis in meat plants and to develop control strategies.

Personal information included in the questionnaire will be treated in confidence and will not be published or disclosed to any third parties (for example your employer) by the research team in a manner that would allow identification of participants.

Date of interview: ____/____/2011 Interviewer's name: ______________________

Meat plant: ______________________

Participant Identification

Name & Sir name	
Did you fill in a consent and confidentiality form?	Yes No Please get both forms filled in now
Postal address	
Contact phone number	
Type of location	Rural Lifestyle***** Urban
Date of Birth	(day/month/year) _____/______/______
Gender	Male Female
With which ethnic affiliation do you identify with?	NZ-Maori Pacific Islander NZ-European<br>Asian Other ________________________

Note: ***** e.g., living on the outskirts of town on a property with > 1 acre of land.

Exposure at Work

Current Info: Your Work at the Meat Plant

For how long have you been working for this meat plant (years)?	_________ years
How many months worked from ___2010 - __ 2011? (Have you had a longer break than 6 weeks)	_______months
Did this plant have a seasonal break?	Yes No
If yes, date work started this season	(day/month/year) _____/_____/_____
Which animal species are you exposed to at work (only ask if multispecies plant)?	
Worked for another meat plant from ___2010 to ___ 2011? If yes, where and species slaughtered/processed?	
Do you believe Leptospirosis presents a serious health risk at your work?	No Yes Maybe Don't know
Job description	
In your daily routine, how often do you get in contact with urine, kidneys, urine bladder or with urine contaminated surfaces?	
Do you recall having animal urine land on your eyes or mouth while in your current job?	No Yes Don't know
Work position/<br>Job title	What proportion of your time do you spend in the following locations in the plant? Refer to drawing of plant and put a cross where you usually work. If you usually change your work position, indicate all positions and estimate the percentage of time you spend at each position. Interviewer: assign a number to each position.

Work position at the meat plant (Sheep)

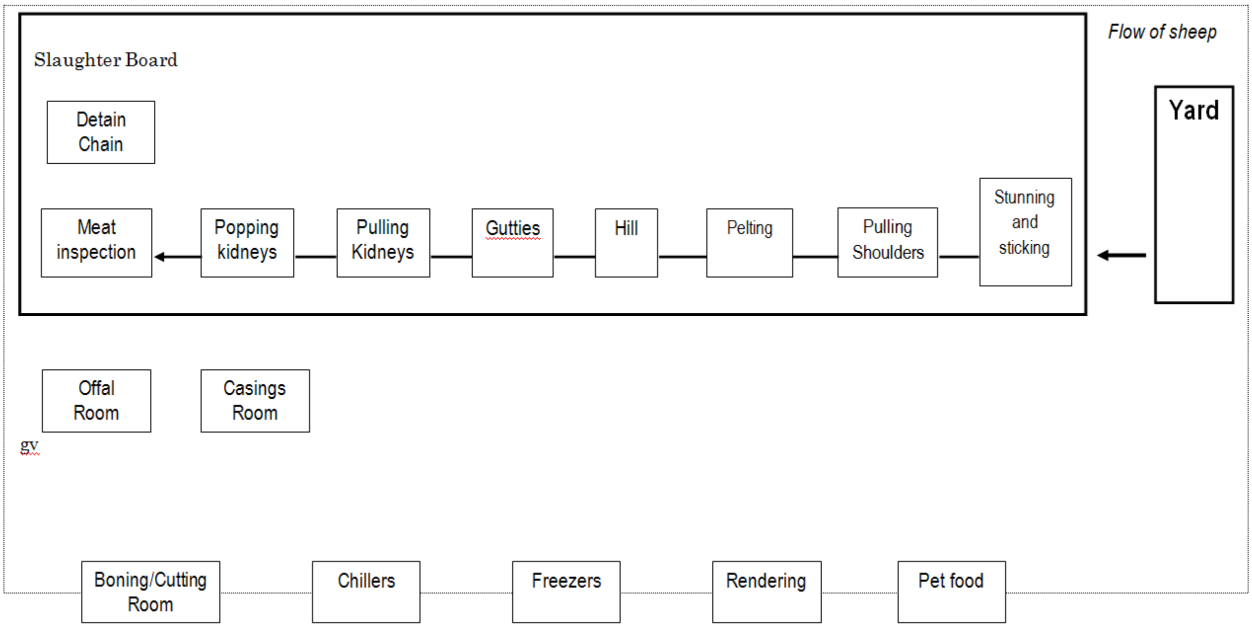

Work position at the meat plant (Beef)

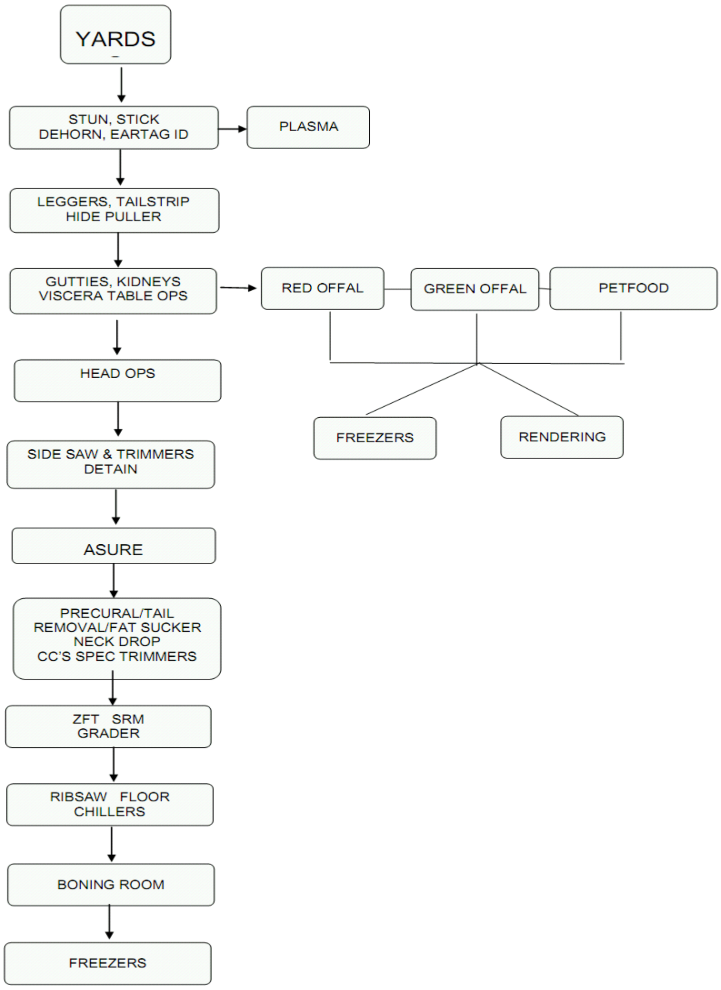

Work Position at the Meat Plant (Deer)

Location	Process Steps	Tick if part of work	Percentage of work time
**Yards**	Receive and pen		
	Ante-mortem inspection		
	Wash		
	Stun		
**Slaughter Floor**	Shackle		
	Stimulate		
	Stick		
	Remove front leg sinew and velvet stubs in season		
	Apply clip to weasand & rod		
	Head work-up		
	Y Cut		
	Belly rip-down, udder, pizzle		
	Clear anus, tail off		
	Tendon and hock removal		
	Remove hide (re-invert to rail) and remove head		
	Evisceration stand: Ring, open belly, eviscerate, trim, popping kidneys		
	Post Mortem Inspection—Inspect head, offal, carcass/retain and re-inspection		
	Carcass dressing check		
	Grading		
	Load carcass to chiller		
**Carcass Chillers**	Chill carcass		
**Boning/Cutting Room**			
**Special purposes offal**	Liver, heart, kidney		
**Special purposes Asia**	Pizzle, tail, sinew, tongue		
**Special purposes blood**			
**Offal Room**			
**Freezers**			
**Pet Food**			
**Office**			
**Other**			
In your daily routine, how often are you using the following protective clothing (PPE) in each work position?	**Face mask with visor down**:<br>Position 1:________________________<br>always often sometimes never<br>Position 2:________________________<br>always often sometimes never<br>Position 3:________________________<br>always often sometimes never<br>**Goggles/glasses:**		

	Position 1:_________________________<br>always often sometimes never<br>Position 2:_________________________<br>always often sometimes never<br>Position 3:_________________________<br>always often sometimes never<br><br>**Balaclava (over face):**<br>Position 1:_________________________<br>always often sometimes never<br>Position 2:_________________________<br>always often sometimes never<br>Position 3:_________________________<br>always often sometimes never
Work position/<br>Job title, continue from last page	**Gloves (any sort):**<br>Position 1:_______________both hands one hand<br>always often sometimes never<br>Position 2:______________ both hands one hand<br>always often sometimes never<br>Position 3:_______________ both hands one hand<br>always often sometimes never
Do you believe Personal Protective Equipment (PPE) will protect you from contracting Leptospirosis?	No Yes Don't know<br>Don't wear any
Do you find wearing PPE inconvenient?	No Yes<br>Don't wear any
Do you smoke at work breaks?	No Yes

Abattoir Work History:

For how long have you been working in an abattoir (years)?	
Have you been working mainly in the meat industry?	Yes No

We Want to Quantify Work Place Exposure for the Last 3 Years

**Year**	**Plant location/ name**	**How many months worked**	**Full time?**	**Species exposure (sheep = S, beef = B, bobbies = BC, deer = D)**	**Positions/ Job titles**
2009/10 (last season)			Yes No		
2008/9 (season before that)			Yes No		
2007/8			Yes No		
Have you worked for an abattoir or butcher before 2007?			Yes No		
If yes, since when?					
If yes, to which animal species were you exposed to?					

Other Regular Work History—Not Abattoir

Over the last 3 years, have you had any other regular work besides your work in an abattoir?

NO Skip to non-work exposures 3.1

YES Complete table below

Type of work	How many hours per week?	Approximately for how long have you done this (years)?	When did you last do this work?
Forestry			
Livestock/ Farming			
Species	Beef cattle Dairy cattle Sheep Goats Deer Pigs		
Horticulture/ cropping/ orchard			
Other			

Non-work Exposures

Regular contact with live animals at home, friend's or family's house

Over the last 3 years, have you had regular (daily or weekly) contact with animals outside work?

NO Skip to **Wildlife Table 3.2**

YES Complete table below

**Animal type**	**No of animals**	**Animals vaccinated against Leptospirosis?**
Beef cattle		No Don't know Yes
Dairy cattle		No Don't know Yes
Sheep		No Don't know Yes
Goats		No Don't know Yes
Deer		No Don't know Yes
Pigs		No Don't know Yes
Dogs		No Don't know Yes
Cats		No Don't know Yes
Other		No Don't know Yes

Wildlife

Over the last 3 years, have you often ***** seen rats, mice, possums, rabbits or hedge hogs at home (house, garden, surrounding fields)?	Yes No Don't know
Do you set traps or poison for these animals at home?	Yes No Don't know

Note: * often: more than or once a week.

Home slaughter

Did you home slaughter or have you helped with home slaughtering any animals in the past 3 years?

NO Skip to **Hunting/Trapping Table 3.4**

YES Complete table below

**Animal type**	**How many per year?**	**How often per year?**	**When was the last time?**
Cattle			
Sheep			
Goats			
Deer			
Pigs			

Hunting/Trapping exposures

Have you been hunting in the last 3 years?

NO Skip to **Other Outdoors Table 3.5**.

YES Complete table below

**Animals hunted**	**For how long have you been doing it (years)?**	**How many shot or trapped in an average year?**	**When shot or trapped an animal last time?**
Deer			
Wild pig			
Small game *****			
Goats			
Other			

Note: ***** e.g., ducks, other birds, possums, rabbits, hares…

Other Outdoor exposures

Over the last 3 years, have you done outdoor activities where you were exposed to fresh water?

NO **Skip to Flooding 3.6**

YES Complete table below

**Outdoor activities *fresh* water**	**For how long have you been doing it (years)?**	**How often per year?**	**When was the last time?**	**Region?**
Camping beside lakes/rivers				
Water sports in lakes/rivers e.g. swimming, boating, windsurfing, endurance events				
Fresh water fishing				
Did you do any of these activities overseas?	No Yes If yes, specify country(ies) ___________			

Flooding

Over the last 3 years has your land been flooded (for several days leaving a water puddle (at least 5m by 10m, 10 cm deep)?

NO **Skip to Previous Illness 4.**

YES When the last time?___________

Previous illness

Have you ever been diagnosed with Leptospirosis?

YES Complete **Lepto Table 4.1**

NO Don't know Skip to **Other illness Table 4.2**

Leptospirosis

Approximate date:	______________	
How was it diagnosed (test)?	Self diagnosed GP Blood test	
Do you know the serovar and/or titre?	Serovar: _______________ Titre:	No
How many days were you off-work or seriously ill?	_________ days	
Please describe the symptoms? *		
Was it treated?	Yes No Don't know/remember<br>Antibiotic treatment If yes, how many days:	
Received ACC compensation?	Yes No Don't know/remember	

Note**:** * e.g., fever, headache, sore muscles or bones, sore eyes, sweating, severe general debility.

Other Illness

Have you had any flu-like symptoms in the last 3 years (*excl. injury*)?	Yes No Approx date ___________	
Have you been off work due to this illness?	# days_________	No
Did you ask for professional help?	GP Nurse Other_____________	No
Were any blood tests done or samples collected?	Yes No NA	
Was a diagnosis made?	Yes , diagnosis of	No Do not remember
Did you have any of the following symptoms?	Fever Headache Sore muscles<br>Sore eyes Sweating Severe debility	
Was it treated?	Yes No Don't know/remember<br>Antibiotic treatment If yes, how many days:	

This is the end of the questionnaire. The research team appreciates your involvement in this study of leptospirosis and is committed to privacy of all personal information.

The lab will check your blood for previous exposure for Lepto and we will notify you of the result by mail as soon as possible."

© 2014 by the authors; licensee MDPI, Basel, Switzerland. This article is an open access article distributed under the terms and conditions of the Creative Commons Attribution license (http://creativecommons.org/licenses/by/3.0/).
